# Oncogenic Roles of Small Nucleolar RNA Host Gene 7 (SNHG7) Long Noncoding RNA in Human Cancers and Potentials

**DOI:** 10.3389/fcell.2021.809345

**Published:** 2022-01-17

**Authors:** Sajad Najafi, Soudeh Ghafouri-Fard, Bashdar Mahmud Hussen, Hazha Hadayat Jamal, Mohammad Taheri, Mohammad Hallajnejad

**Affiliations:** ^1^ Student Research Committee, Department of Medical Biotechnology, School of Advanced Technologies in Medicine, Shahid Beheshti University of Medical Sciences, Tehran, Iran; ^2^ Department of Medical Genetics, School of Medicine, Shahid Beheshti University of Medical Sciences, Tehran, Iran; ^3^ Department of Pharmacognosy, College of Pharmacy, Hawler Medical University, Erbil, Iraq; ^4^ Center of Research and Strategic Studies, Lebanese French University, Erbil, Iraq; ^5^ Department of Biology, College of Education, Salahaddin University-Erbil, Erbil, Iraq; ^6^ Institute of Human Genetics, Jena University Hospital, Jena, Germany; ^7^ Skull Base Research Center, Loghman Hakim Hospital, Shahid Beheshti University of Medical Sciences, Tehran, Iran

**Keywords:** SNHG7, non-coding RNA, lncRNA, cancer, biomarker

## Abstract

Long noncoding RNAs (lncRNAs) are a class of noncoding transcripts characterized with more than 200 nucleotides of length. Unlike their names, some short open reading frames are recognized for them encoding small proteins. LncRNAs are found to play regulatory roles in essential cellular processes such as cell growth and apoptosis. Therefore, an increasing number of lncRNAs are identified with dysregulation in a wide variety of human cancers. SNHG7 is an lncRNA with upregulation in cancer cells and tissues. It is frequently reported with potency of promoting malignant cell behaviors *in vitro* and *in vivo*. Like oncogenic/tumor suppressor lncRNAs, SNHG7 is found to exert its tumorigenic functions through interaction with other biological substances. These include sponging target miRNAs (various numbers are identified), regulation of several signaling pathways, transcription factors, and effector proteins. Importantly, clinical studies demonstrate association between high SNHG7 expression and clinicopathological features in cancerous patients, worse prognosis, and enhanced chemoresistance. In this review, we summarize recent studies in three eras of cell, animal, and human experiments to bold the prognostic, diagnostic, and therapeutic potentials.

## Introduction

Initially based on the central dogma of molecular biology lasting for decades, sequential flow of cell genetic information was defined through RNAs, which encoded proteins, and so messenger RNA (mRNA) were considered mediators of template DNA and downstream proteins (Crick, 1970). However, exceptions were gradually made, and RNAs that did not directly encode any protein or polypeptide were identified. Transfer RNAs (tRNA), ribosomal RNAs (rRNAs), and small nuclear and nucleolar RNAs (snRNAs and snoRNAs, respectively) were recognized as groups of non-protein-coding RNAs (ncRNAs) with functions in the translation of coding mRNAs and modification or processing of other RNAs (Hombach and Kretz, 2016). Nowadays, we know that a minority of large genomes in complex eukaryotic organisms encode protein or polypeptide strands, and a majority [for instance, 98% in humans (Elgar and Vavouri, 2008)] does not encode for amino acids. This great proportion, formerly called “junk DNA,” however, is mainly [e.g., two thirds of the mammalian genomes (Mattick, 2001)] transcribed to thousands of RNA transcripts, including various types of known ncRNAs that are demonstrated to be involved in critical cellular processes through conducting regulatory functions (Najafi et al., 2022). By employment of high-throughput technologies, such as RNA-seq, identification of novel ncRNAs is accelerated, and new members are being introduced constantly (Taheri et al., 2021). Although the role of ncRNAs is not yet clear, however, their involvement in essential life processes have caused them to be the architects of complexity in eukaryotes (Mattick, 2001). The number of functional ncRNAs are growing, and several show regulatory roles on gene expression.

The size of the transcript is the main discriminating parameter used for classification of ncRNAs. Based on a size limit, ncRNAs are divided in two short and long classes. MicroRNAs (miRNAs), rRNAs, tRNAs, and snRNAs/snoRNAs are several described subclasses of short ncRNAs with a total length shorter than 200 nucleotides (Amin et al., 2019). Among them, miRNAs are studied more broadly compared with others, an increasing number identified in mammalian cells, and also a number are reported with altered expression in various human diseases.

Long noncoding RNAs (lncRNAs) are the second class of ncRNAs with characteristic length of >200 nucleotides. Thousands of lncRNA-related genes have been identified in the human genome and corresponding transcripts reported in large quantities by a large number ranging from 10,000 to 60,000 in human cells (Guttman et al., 2009; Iyer et al., 2015). They have been identified in a wide variety of eukaryotic species, and several show conserved sequences among different organisms suggesting evolution pressure (Ramírez-Colmenero et al., 2020). A number of exclusive properties have made lncRNAs different compared with regular mRNAs. These remarkable differences include characteristic biogenesis, localization, structure, and roles (Quinn and Chang, 2016). Unlike protein-coding RNAs, lncRNAs are mainly transcribed from regulatory and noncoding sequences such as promotors, enhancers, and introns. Furthermore, they could be generated from shared sequences with other transcripts (Al-Tobasei et al., 2016) although some researchers consider lncRNAs as noises or byproducts of transcription (Gao et al., 2020). Unlike their names, some short open reading frames are recognized for them that encode for small proteins (Hartford and Lal, 2020). According to the location of transcription, lncRNAs are classified into intronic and intergenic. Structurally, lncRNAs can be found in linear and circular forms, which are mainly referred to as the former structures; however, circular RNAs also have been found with regulatory functions and roles in pathogenesis of various human cancers (Rahmati et al., 2021; Sayad et al., 2021). LncRNAs show specific expression in cell-, tissue-, and developmental stage–specific manners (Sarropoulos et al., 2019). Their biogenesis is also forced to more strict regulation relative to protein-coding transcripts that, along with their conservation among species, suggests critical regulatory functions for lncRNAs (Dahariya et al., 2019). Several strategies, including ribonuclease P cleavage, processing by ribonucleoproteins, and circularization via backsplicing, play a role in biogenesis of lncRNAs (Dahariya et al., 2019). Same as mRNAs, lncRNAs undergo post-transcriptional modifications on processing such as capping and polyadenylation at 5ˊ and 3ˊ ends, respectively, splicing and base modifications (Sarropoulos et al., 2019). They are mainly located at the nucleus exerting their epigenetic and gene expression regulatory functions via altering the histone modifications or transcription control through several mechanisms, including scaffold, signal, guide, and decoy (Zhang et al., 2019a; Dahariya et al., 2019). Through these ways, lncRNAs in interactions with DNA, proteins, and other RNAs, play a role in various biological phenomena, such as cell differentiation and reprogramming, organ development, immune responses, and cell cycle control (Statello et al., 2021).

Accordingly, a set of lncRNAs is found to be deregulated in various human disorders. An association between expression level of these transcripts and pathogenesis in major health conditions confirms critical roles of lncRNAs in essential health-affecting processes. Among an increasing number of pathogenic lncRNAs, a handful, such as XIST, MALAT1, HOTAIR, H19, ANRIL, and MEG3, are the best known and most often found transcripts. Playing a role particularly in cancer development and progression, differentially expressed lncRNAs in cancer tissues are functionally subdivided into two group of oncogenic and tumor suppressors.

Small nucleolar RNA host gene 7 (SNHG7) is among the oncogenic lncRNAs with progressive effects in multiple human cancers although a single study suggests tumor suppressor function for SNHG7 in pituitary adenoma (Xue and Ge, 2020). Its corresponding gene, located on chromosome 9q34.3, encodes a 2157-base-pair-long transcript. SNHG7 was reported for the first time in 2013 by Chaudhry in X-ray-treated lymphoblastoid cells (Chaudhry, 2013). Rather than regulation of transcription factors, translation, or stability of mRNAs involved in several diseases such as cardiac fibrosis, hepatic fibrosis, and cardiac hypertrophy in addition to helping fracture repair (Chen et al., 2019a; Jing et al., 2019; Yu et al., 2019; Wang et al., 2020a), SNHG7 is found to be overregulated in cancer tissues compared with healthy tissues in a wide variety of human malignancies, including bladder, prostate, gastric, colorectal, and pancreatic cancers (Li et al., 2018a; Zhong et al., 2018a; Cheng et al., 2019a; Han et al., 2019; Zhang et al., 2020a). This upregulation also is demonstrated to accelerate cancer progression. It is shown that SNHG7 is negatively regulated by insulin-like growth factor 1 (IGF1) signaling at the post-transcriptional level through the MAPK pathway to control cell proliferation (Boone et al., 2020). In cell and animal studies, SNHG7 is shown with oncogenic roles in accordance with clinicopathological features and also diagnostic and prognostic values in cancerous patients. In this review, we have gathered recent findings on the oncogenic roles of this lncRNA in three levels of cell, animal, and human studies with a focus on clinical results predicting SNHG7 as a novel biomarker for different types of human cancers.

### Cell Line Studies

**FIGURE 1:**
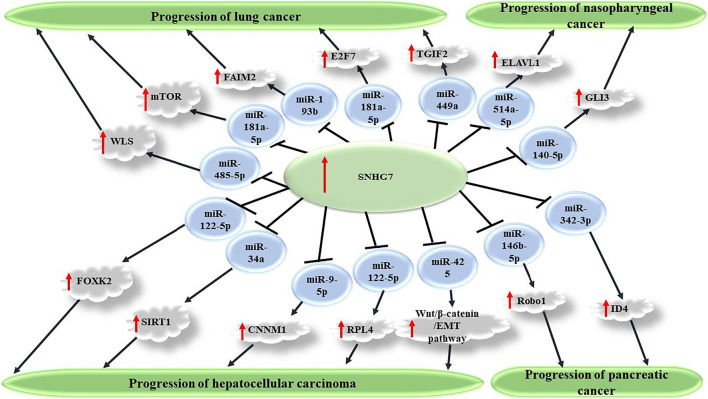
SNHG7 promotes carcinogenesis via sponging miRNAs and consequently upregulating several transcription factors. Through repression of target miRNAs, including miR-449a, miR-181a-5p, miR-193b, and miR-485-5p, SNHG7 causes increased expression of TGIF2, mTOR, FAIM2, and WLS factors, which consequently promote malignant features of cancer cells and enhanced carcinogenesis in non-small cell lung carcinoma. SNHG7 also plays a role in progression of several other cancers, including nasopharyngeal, pancreatic, and hepatocellular carcinoma.

**FIGURE 2:**
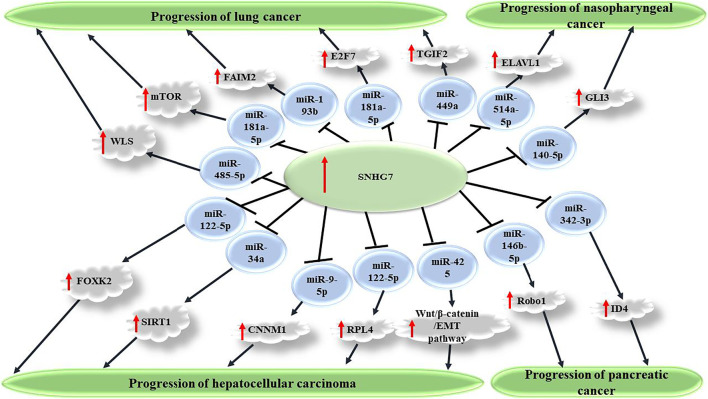
Signaling pathways and cellular processes are affected by SNHG7 to enhance tumor progression in cancer cells. Through sponging miR-34a, SNHG7 activates several signaling pathways, such as Notch-1 and PI3K/Akt/mTOR in breast and colorectal cancer, respectively. Other pathways, such as Wnt/b-catenin and K-ras/ERK/cyclinD1, are also affected via other target miRNAs. The effect of SNHG7 on enhancement of glycolysis through upregulation of lactate dehydrogenase A (LDHA) has beneficial effects for cancer cells metabolism.

Through study of SNHG7 knockdown or overexpression in cancer cell lines, it is demonstrated that expression of this oncogenic lncRNA promotes malignant features of the cells *in vitro*. This universal finding, although opposite effects have been described for SNHG7 at least in two distinct experiments (Pei et al., 2020; Huang et al., 2020), is reported for a broad spectrum of cancer cell types, such as breast, colorectal, bladder, gastric, liver, etc. Proliferation and colony formation experiments have unveiled increased cell and colony numbers in cancer cells in response to SNHG7 simulated excess expression compared with baseline conditions. Accordingly, reduced apoptosis consistent with elevated tumor cell growth hypothesizes the role of this lncRNA in cancer progression. Migratory and invasive potentials of cancer cells also show enhancement in Transwell and Matrigel assays, respectively. Conferring chemoresistance or desensitization has been concluded from cellular studies in which increased sensitivity of cancer cells to conventional chemotherapy agents and/or radiotherapy is seen on SNHG7 knockdown. For example, in two distinct experiments, enhanced sensitivity of breast cancer cells to Adriamycin and Trastuzumab is shown when SNHG7 is silenced or its target sponged miRNA (miR-34a or miR-186, respectively) is overexpressed (Li et al., 2020a; Zhang et al., 2020b). Knockdown studies employing RNA silencing confirm the overexpression experiment results by reversing the SNHG7 impacts on malignant cells behaviors. Via making a network, lncRNAs are known to affect expression of a specific target miRNA. Dual luciferase reporter and RNA immunoprecipitation (RIP) assays confirm the association between SNHG7 and target miRNA consistent with bioinformatics predictions. These interactions seem to be conducted via complementary sequences as binding sites on miRNA for SNHG7. This regulatory effect is mainly repressive, and expression levels in quantitative real-time polymerase chain reaction (qRT-PCR) reveal a negative correlation between both. It is hypothesized that through downregulation of the target miRNA, SNHG7 as a competing endogenous RNA (ceRNA) or sponger exerts its regulatory impacts on downstream transcription factors playing a role in some signaling pathways (Figure 1). Activation of an oncogenic signaling pathway demonstrates why these lncRNAs are considered to have tumor promoting potentials. A handful of evidence on the acceleration of the cell cycle in response to SNHG7 overexpression or arrest in a phase under knockdown conditions suggests indirect enhancing influences of this lncRNA on cell proliferation and differentiation, which consequently, leads to cancer progression. For instance, She et al. (2018) find that SNHG7 upregulates the Fas apoptotic inhibitory molecule 2 (FAIM2) through sponging miR-193b in non-small cell lung cancer (NSCLC) cells. *In silico* investigations demonstrate binding sites for miR-193b on the SNHG7 sequence. FAIM2 is a membrane protein; shows antiapoptotic activity; is upregulated in several cancers; and is already known to promote tumor cell proliferation, migration, and invasion in lung cancer cells (She et al., 2016). Repression of proapoptotic proteins, such as Bax, and SIRT1-associated pyroptosis also benefits reduced tumor cell death (Xu et al., 2019; Chen et al., 2020a). Thus, it is not surprising to see repressed apoptosis frequently reported on SNHG7 overexpression, which means steady growth of cancer cells. Enhanced glycolysis through upregulation of lactate dehydrogenase A (LDHA) in the tumor microenvironment is another finding on SNHG7 overexpression, which can help the cancer cell economy (Zhang et al., 2019b; Pei et al., 2021). SNHG7 also causes arrest in the G1/G0 phase of the cell cycle (Wang et al., 2017a; Xu et al., 2018); regulates signaling pathways, such as Wnt/β-Catenin and AKT/mTOR pathways; and represses tumor suppressors, such as P15 and P16 (Wang et al., 2017b; Li et al., 2020b; Chi et al., 2020; Du et al., 2020). Furthermore, an elevated neovascularization rate following SNHG7 overexpression is consistent with tumor progression conditions (Li et al., 2018b). Playing a role in regulation of cellular processes, signaling pathways, transcription factors, and particularly via sponging miRNAs, SNHG7 is described as an oncogenic lncRNA with upregulation in various types of cancer cells (Figure 2).

### Animal Studies

Xenograft animal experiments with inoculation of cancer cells into nude mice try to simulate the cancer conditions in an animal model. BALB/C nude mice are used to evaluate the effect of lncRNA upregulation and/or downregulation *in vivo*. Cancer cells transfected with a vector expressing small heterogenous RNA (shRNA) for overexpression or small interfering RNA for knockdown of the lncRNA along with a vector expressing a control scrambled sequence are injected into the flank of nude mice to establish the xenograft mouse model. Size and volume of the tumor created in the mice is then calculated to compare the tumor growth after sacrificing the animals. Using immunohistochemistry for detection of Ki-67 as a proliferation marker in excised tumor tissues, it is feasible to assess the implanted tumor cell proliferation. Xenograft animal experiments in a number of studies demonstrate that SNHG7 knockdown suppresses tumor growth via decreasing tumor size *in vivo*, whereas faster tumor growth is reported for SNHG7-overexpressing implanted cells compared with control animals. This effect is reported for SNHG7 silencing in various cancer models (Table 1). Decreased tumor metastasis or repression of some carcinogenic signaling pathways, such as the Notch pathway, is also reported in other animal studies (Sun et al., 2019). For instance, several studies assess the role of SNHG7 knockdown on hepatocellular carcinoma (HCC) growth in xenograft mice (Yang et al., 2019; Yao et al., 2019; Xie et al., 2020; Zhao et al., 2021). Yang et al. (2019) demonstrate lower tumor volume and percentage of Ki-67-stained HCCLM3 cells and less lung metastasis of HCCLM3 cells in an SNHG7 knockdown mice group compared with the control group. Additionally, Yao et al. (2019) show that the expression of the metastasis-associated protein matrix metalloproteinase-9 (MMP-9) is increased in SNHG7-overexpressing HepG2 implanted cells, suggesting a mechanism for enhancing the effect of SNHG7 on tumor metastasis. Collectively, promoted tumor growth on SNHG7 overexpression and/or suppressed tumor proliferation on SNHG7 knockdown is reported in a body of studies. These results, along with cellular findings, confirm the oncogenic role of SNHG7, and the knockdown achievements may suggest therapeutic potentials for anticancer therapies.

**TABLE 1:** Effects of SNHG7 on tumor growth and metastasis in animal studies.

Cancer type	Animal models	Function	References (s)
Pancreatic cancer	Nude mice	Δ SNHG7: ↓ tumor growth	Jian and Fan, (2021)
	Female BALB/C nude mice	Δ SNHG7: ↓ tumor growth	Cheng et al. (2019b)
Breast cancer	BALB/c nude mice	Δ SNHG7: ↓ tumor growth	Zhang et al. (2020b)
	BALB/c nude mice	Δ SNHG7: ↓ tumor growth	Li et al. (2020d)
	BALB/c athymic nude mice	Δ SNHG7: ↓ tumor growth, ↓EMT, and ↓Notch-1 pathway	Sun et al. (2019)
Colorectal cancer	Nude mice	Δ SNHG7: ↓ tumor growth	Li et al. (2018b)
Lung cancer (non-small cell lung cancer; NSCLC)	Nude mice	Δ SNHG7: ↓ tumor growth	Li et al. (2020b)
	Athymic nude mice	Δ SNHG7: ↓ tumor growth	Wang et al. (2020b)
	Nude mice	Δ SNHG7: ↓ tumor growth	She et al. (2018)
Liver cancer (hepatocellular carcinoma; HCC)	BALB/c male nude mice	Δ SNHG7: ↓ tumor growth	Zhao et al. (2021)
	BALB/c male nude mice	Δ SNHG7: ↓ tumor growth	Xie et al. (2020)
	BALB/c nude mice	Δ SNHG7: ↓ tumor growth, and ↓metastasis	Yang et al. (2019)
	BALB/c nude mice	Δ SNHG7: ↓ tumor growth	Yao et al. (2019)
Gastric cancer	BALB/c mice	Δ SNHG7: ↓ tumor growth	Wang et al. (2017b)
Bladder cancer	Male nude mice	Δ SNHG7: ↓ tumor growth	Wang et al. (2020c)
Pituitary adenocarcinoma	Nude mice	Δ SNHG7: ↓ tumor growth	Yue et al. (2021)
Neuroblastoma	BALB/c nude mice	Δ SNHG7: ↓ tumor growth	Jia et al. (2020)
Glioma	BALB/c nude mice	Δ SNHG7: ↓ tumor growth	Du et al. (2020)
Thyroid	BALB/c nude mice	Δ SNHG7: ↓tumor cell proliferation, and ↓^131^I resistance	Chen et al. (2021)
Glioblastoma	BALB/c nude mice	Δ SNHG7: ↓ tumor growth, and ↓metastasis	Ren et al. (2018)
Ovarian cancer	BALB/c nude mice	Δ SNHG7: ↓ tumor growth	Bai et al. (2020)
Cervical cancer	BALB/c nude mice	Δ SNHG7: ↓ tumor growth	Zhao et al. (2020)
Prostate cancer	BALB/c nude mice	Δ SNHG7: ↓ tumor growth, and ↑cell cycle arrest	Qi et al. (2018)

### Human Studies

Consistent with cellular findings, enormous expression assessments using qRT-PCR analysis demonstrate elevated SNHG7 expression in tissues retrieved from cancerous patients compared with healthy adjacent tissues. Increased SNHG7 tissue expression is frequently found to be associated with worse clinicopathological features, which are used in clinical classification and staging of human malignancies. Importantly, patients with more advanced clinicopathological characteristics are predicted to have worse prognosis and severe outcomes. These include larger tumor size, more advanced clinical stage, poor histologic grade, deeper tumor invasion, and lymph node metastasis in accordance with high SNHG7 expression in the affected patients (Zeng et al., 2019; Pang et al., 2020; Zhu et al., 2021). This value is also shown in malignancies with broad and different features and in meta-analyses pooling data of tens of studies (Yu et al., 2021a; Yi et al., 2021; Yu et al., 2021b). For example, in acute myeloid leukemia (AML), an association between SNHG7 and SNHG12 lncRNAs and specific clinical/molecular features, including white blood cell (WBC) counts and mutations in *IDH1*, *RUNX1*, and *NPM1* genes, shows high value of SNHG7 in correlation with extensive features (Shi et al., 2020). These demonstrations suggest that elevated SNHG7 expression predicts poor clinicopathological characteristics. In other words, high SNHG7 expression can predict worse outcomes following poor clinicopathological determinants. In accordance with clinicopathological findings, SNHG7 also shows correlation with prognostic parameters. Survival analysis using a Kaplan–Meier curve indicates shorter survival time in overall survival (OS) and disease-free survival (DFS) for patients with high SNHG7 expression relative to those with low levels. This finding is reported for various human cancers, for which survival analysis is conducted (Table 2). For example, in three distinct studies that reported survival analyses in HCC patients, among a total of 150 patients, poorer OS time was reported separately for the patients with elevated tissue SNHG7 expression in comparison to those with low levels (Yang et al., 2019; Zhao et al., 2021; Yao et al., 2019). Additionally, recurrence is predicted to happen in shorter durations and higher rates among patients with high SNHG7 expression (Zhang et al., 2020c). Interestingly, Cox regression analyses confirm the predictive value of SNHG7 as an independent prognostic factor among cancerous patients. This is particularly reported in several district experiments on human malignancies such as gastric cancer, cervical cancer, HCC, and liver metastasis following hepatectomy in CRC patients (Table 2) (Zeng et al., 2019; Zhang et al., 2020a; Zhang et al., 2020c; Shen et al., 2020). As for diagnostic values, an area under curve (AUC) of 0.84 in the receiver operating characteristic (ROC) curve is reported for SNHG7 in CRC patients (Hu et al., 2019). Importantly, SNHG7 is demonstrated as a potential therapeutical target as it is identified in several studies to lead to enhanced chemoresistance to several anticancer agents such as Cisplatin, Trastuzumab, and Folfirinox in the cancer cells (Chen et al., 2019d; Li et al., 2020a; Zhang et al., 2020b; Dai et al., 2020; Cheng et al., 2021; Pei et al., 2021). Also, metformin with anticancer properties is found to exert its effects in sensitization to Paclitaxel via regulation of SNHG7/miR-3127-5p-mediated autophagy in ovarian cancer cells (Yu et al., 2020). In another study, metformin is demonstrated to suppress growth of hypopharyngeal cancer cells through epigenetic silencing of SNHG7 (Wu et al., 2019). Taken together, human studies suggest SNHG7 lncRNA with promising diagnostic, prognostic, and therapeutic potentials in various types of cancer.

**TABLE 2:** Clinical prognostic importance of SNHG7 in human cancers.

Cancer type	Clinical samples	Expression change in tumor tissues compared to normal tissues	Associated clinical features	Kaplan–Meier analysis	Multivariate cox regression	References (s)
Lung cancer	36 cancerous patient tissues and matched NATs	Upregulated	—	Patients with elevated expression levels of SNHG7 demonstrated decreased OS rate compared to those with lower levels	—	Li et al. (2020b)
	30 cancerous patient tissues and matched NATs	Upregulated	—	—	—	Wang et al. (2020b)
Esophageal cancer	40 cancerous patient tissues and matched NATs	Upregulated	—	—	—	Wang et al. (2021)
Liver (hepatocellular carcinoma; HCC)	30 cancerous patient tissues and matched NATs	Upregulated	Tumor size, TNM grade, and Distant metastasis	Log-rank test demonstrated that patients with high SNHG7 expression had poorer OS.	—	Zhao et al. (2021)
	25 cancerous patient tissues and matched NATs	Upregulated	—	—	—	Chen et al. (2020a)
	80 cancerous patient tissues and matched NATs	Upregulated	Tumor stages, tumor grade, and vascular invasion	Patients with high SNHG7 expression levels had poor OS.	—	Yang et al. (2019)
	40 cancerous patient tissues and matched NATs	Upregulated	TNM stage, and tumor metastasis	Elevated SNHG7 expression was markedly associated with poor OS in hepatic carcinoma patients	—	Yao et al. (2019)
	100 cancerous patient tissues and matched NATs	Upregulated	Tumor number, lymph node metastasis, and clinical stage	Patients with high SNHG7 expression demonstrated worse OS and PFS relative to those with low levels	SNHG7 expression acts as an independent prognostic factor in HCC patients	Shen et al. (2020)
Synchronous colorectal liver metastasis (SCLM)	96 SCLM patients	Upregulated	Differentiation of primary tumor, invasion depth of primary focus, lymph node metastases, number of liver metastases, and liver metastasis grade	Patients with high SNHG7 expression levels had poor OS.	SNHG7 expression acts as an independent prognostic factor for OS and occurrence in SCLM patients	Zhang et al. (2020c)
Pancreatic cancer	50 cancerous patient tissues and matched NATs	Upregulated	tumor size, TNM stage, lymph node metastasis, and distant metastasis	Patients with elevated expression levels of SNHG7 demonstrated decreased survival rate relative to those with lower levels	—	Jian and Fan, (2021)
	40 cancerous patient tissues and matched NATs	Upregulated	—	Patients with high SNHG7 expression levels had poor OS.	—	Cheng et al. (2019b)
Breast cancer	43 cancerous patient tissues	Upregulated	Tumor size, TNM stage, and Ki-67 index	Patients with high SNHG7 levels had lower DFS compared to those with lower levels	—	Li et al. (2020a)
	50 cancerous patient tissues and matched NATs	Upregulated	Pathological stage, and lymph node metastasis	—	—	Li et al. (2020d)
	837 cancerous patient tissues and matched NATs	Upregulated	—	High SNHG7 was associated with decreased survival in breast cancer patients	—	Zhang et al. (2019b)
	72 cancerous patient tissues and matched NATs	Upregulated	Clinical Stage, lymph node and distant metastasis	High SNHG7 was correlated with shorter survival time in breast cancer patients	—	Luo et al. (2018)
Gastric cancer	30 cancerous patient tissues and 30 healthy tissues	Upregulated	—	—	—	Pei et al. (2021)
	36 cancerous patient tissues and matched NATs	Upregulated	—	—	—	Zhao and Liu, (2021)
	162 cancerous patient tissues and matched NATs	Upregulated	TNM stage, depth of invasion, lymph node and distant metastasis	Patients with high SNHG7 levels showed lower OS compared to those with high SNHG7 expression	SNHG7 acts as an independent factor for poor OS in patients with gastric cancer	Zhang et al. (2020a)
Bladder cancer	60 cancerous patient tissues and matched NATs	Upregulated	Clinical stage	Patients with high SNHG7 levels showed unfavorable prognosis	—	Wang et al. (2020c)
	92 cancerous patient tissues and matched NATs	Upregulated	Tumor range, lymph nodes, and pathological stage	Patients with high SNHG7 levels had poor OS compared to those with low levels	—	Chen et al. (2019b)
Pituitary adenocarcinoma	30 cancerous patient tissues and matched NATs	Upregulated	—	Patients with high SNHG7 levels showed unfavorable prognosis compared to those with low levels	—	Yue et al. (2021)
Glioma	30 cancerous patient tissues and matched NATs	Upregulated	—	—	—	Cheng et al. (2020)
	20 and 33 cancerous patient tissues and matched NATs	Upregulated	Tumor grade	—	—	(Du et al., 2020; Deng et al., 2021)
Glioblastoma	53 cancerous patient tissues and matched NATs	Upregulated	WHO Grade	--	--	Chen et al. (2020b)
	53 cancerous patient tissues and matched NATs	Upregulated	—	Patients with high SNHG7 levels had poor survival rates compared to those with low levels	—	Ren et al. (2018)
Neuroblastoma	45 cancerous patient tissues and matched NATs	Upregulated	Clinical stage	Patients with low SNHG7 levels demonstrated longer OS compared to those with high levels	—	Jia et al. (2020)
	92 cancerous patient tissues and matched NATs	Upregulated	Lymph node metastasis, INSS stage, and optic nerve invasion	Patients with high SNHG7 levels had poorer prognosis compared to those with high levels	—	Chi et al. (2019)
Thyroid cancer	56 normal samples and 578 tumor samples	Upregulated	Pathology stage	Patients with high SNHG7 levels shorter DFS times compared with those with low levels	—	Chen et al. (2019c)
Cervical cancer	45 cancerous patient tissues and matched NATs	Upregulated	Tumor Size, FIGO Stage, and lymph-Node Metastasis	Patients with high SNHG7 levels demonstrated poorer OS compared with those with low levels	—	Zhao et al. (2020)
	60 cancerous patient tissues and matched NATs	Upregulated	TNM stage, lymph node metastasis, and depth of tumor invasion	Patients with high SNHG7 levels demonstrated poorer OS compared with those with low levels	SNHG7 acts as an independent factor for poor OS in patients with gastric cancer	Zeng et al. (2019)
Colorectal cancer	48 cancerous patient tissues and matched NATs	Upregulated	Clinical stage, lymph node and distant metastasis	High SNHG7 expression was correlated with poor survival	—	Shan et al. (2018)
	198 cancerous patient tissues and matched NATs	Upregulated	Invasion depth	High SNHG7 expression was correlated with poor OS.	SNHG7 expression is an independent prognostic risk factor for OS in CRC patients	Hu et al. (2019)
Prostate cancer	499 cancerous patient tissues and matched NATs	Upregulated	—	—	—	Han et al. (2019)
	42 cancerous patient tissues and matched NATs	Upregulated	Gleason score, and tumor stage	Patients with high SNHG7 expression had poor OS compared to those with low expression	—	Qi et al. (2018)
	127 cancerous patient tissues and matched NATs	Upregulated	TNM stage, Gleason score, bone, and pelvic lymph node metastasis	Patients with high SNHG7 expression had poor prognosis compared to those with low expression	SNHG7 acts as an independent factor for poor prognosis in patients with prostate cancer	Xia et al. (2020)
Osteosarcoma	30 cancerous patient tissues and matched NATs	Upregulated	Tumor size, high Enneking staging, and distant metastasis	Patients with high SNHG7 levels had shorter survival time compared with those with low levels	—	Deng et al. (2018)
Chromophobe renal cell carcinoma	Tissue expression of 59 patients retrieved from the TCGA database and 23 NATs	Upregulated	—	SNHG7 level was associated with OS	—	He et al. (2016)

OS: overall survival, DFS: disease-free survival, PFS: progression-free survival.

## Discussion

LncRNAs are a group of ncRNA transcripts defined with a length of >200 nucleotides. Although not elucidated, however, a number of regulatory functions are described for lncRNAs. They are involved in controlling several biological processes, such as cell cycle and proliferation. Accordingly, dysregulation of lncRNAs is identified in a number of human malignancies, suggesting diagnostic and therapeutic potentials. SNHG7 is an lncRNA that has been studied as an oncogenic transcript in a handful of cellular and animal experiments. It is upregulated in cancer cells and tissues retrieved from cancerous patients. SNHG7 is shown to be predominantly localized in the cytoplasm, where it serves as a ceRNA to sponge miRNAs and control expression of downstream targets (Hu et al., 2020). *In vitro* experiments frequently demonstrate a promoted malignant phenotype of cancer cells on SNHG7 overexpression, whereas its knockdown reverses tumor cell proliferation, migration, and invasion and enhances apoptosis. These regulatory effects are thought to be conducted through an axis of action affecting translation and stability of several transcription factors and signaling pathways mediated by sponging target miRNAs. Not a single one, but plenty of miRNAs are identified to be sponged by SNHG7 (see Table 3). Xenograft animal studies confirm the oncogenic role of SNHG7 as tumor growth and metastasis of grafted cancer cells are promoted, whereas SNHG7 knockdown represses them (see Table 1). For a reported association between upregulated SNHG7 expression and worse clinicopathological characteristics in cancerous patients, clinical studies support oncogenic features of SNHG7. Eventually, Kaplan–Meier survival and Cox univariate and multivariate analyses suggest SNHG7 as a potential prognostic and diagnostic biomarker for human malignancies. Importantly, knockdown experiences and also the contributing role of SNHG7 in chemoresistance suggest it as a potential therapeutic target, which can benefit the anticancer therapies.

**TABLE 3:** An overview to the oncogenic influences of SNHG7 in cell studies of different types of cancer.

Cancer type	Targets/Regulators and signaling pathways	Assessed cell lines	Function	References(s)
Lung cancer	miR-485-5p/WLS axis	H1650, H1975, A549 and H1299	Δ SNHG7: ↓tumor cell proliferation, ↓migration, and ↓invasion	Li et al. (2020c)
	miR-181a-5p/AKT/mTOR axis	A549, and NCI-H1299	Δ SNHG7: ↓tumor cell proliferation, ↓migration, ↓invasion and ↑apoptosis	Li et al. (2020b)
	miR-193b/FAIM2 axis	Beas‐2B, H125, 95D, and A549	↑↑ SNHG7: ↑↑FAIM2: ↑tumor cell proliferation, ↑migration, and ↑invasion	She et al. (2018)
	miR-181a-5p/E2F7 *Axis*	NCI-H520, SPC-A1, H-23, and BEAS-2B	Δ SNHG7: ↓tumor cell viability, ↓colony formation, ↓migration, ↓invasion and ↑apoptosis	Wang et al. (2020b)
	miR-449a/TGIF2 axis	BEAS-2B, A549, and H1299	Δ SNHG7: ↓tumor cell proliferation, ↓migration, ↓invasion, and ↓EMT	Pang et al. (2020)
	FAIM2	BEAS-2B, H125, 95D, and A594	Δ SNHG7: ↓tumor cell proliferation, ↓migration, ↓invasion and ↑apoptosis	She et al. (2016)
	miR-34a-5p	NSCLC cells	↑↑ SNHG7: ↑tumor cell proliferation	Chai et al. (2021)
Esophageal cancer	miR-625/SNHG7 axis	TE1, EC109, TE13, and YES2	Δ SNHG7: ↓tumor cell proliferation, ↓migration, and ↓invasion	Wang et al. (2021)
	—	HEEC, Eca109, EC9706, TE-10, and TE-11	Δ SNHG7: ↓tumor cell proliferation, ↑cell cycle arrest, and ↑apoptosis	Xu et al. (2018)
Nasopharyngeal cancer	miR-514a-5p/ELAVL1 axis	NP69, CNE1, CNE2, C666–1 and HNE1	↑↑ SNHG7: ↑tumor cell proliferation, and ↑colony formation	Hu et al. (2020)
	miR-140-5p/GLI3 axis	CNE1, HONE1, C666-1, and CNE2	Δ SNHG7: ↓tumor cell proliferation, ↓colony formation, ↓drug resistance, and ↑apoptosis	Dai et al. (2020)
Liver cancer (hepatocellular carcinoma; HCC)	miR-122-5p/FOXK2 axis	SNU449, Hep3B, and THLE-2	Δ SNHG7: ↓tumor cell proliferation, ↓migration, ↓invasion, and ↓EMT	Zhao et al. (2021)
	miR-34a/SIRT1 axis	THLE-3, HEK-293, HepG2, and SK-hep-1	Δ SNHG7: ↑NLRP3-dependent pyroptosis	Chen et al. (2020a)
	miR-9-5p/CNNM1 axis	THLE-3, BEL-7404, HCCLM3, Hep3B and HepG2	Δ SNHG7: ↓tumor cell proliferation, ↓colony formation, and ↑apoptosis	Xie et al. (2020)
	miR-122-5p/RPL4 axis	Hhu7, Hep3B, HCCLM3, and MHCC97H	Δ SNHG7: ↓tumor cell proliferation, ↓migration, and ↓invasion	Yang et al. (2019)
	miR‐425/Wnt/β‐catenin/EMT pathway	HepG2, and HCC-LM3	Δ SNHG7: ↓tumor cell proliferation, ↓migration, and ↓invasion	Yao et al. (2019)
Pancreatic cancer	miR-146b-5p/Robo1 axis	PANC-1, SW 1990, BxPC-3 and AsPC-1	Δ SNHG7: ↓tumor cell proliferation, ↓migration, ↓invasion and ↑apoptosis	Jian and Fan, (2021)
	miR-342-3p/ID4 axis	HPDE6-C7, HEK293T, AsPC-1, BxPC-3, SW 1990, PANC-1, and PaCa-2	Δ SNHG7: ↓tumor cell proliferation, ↓migration, and ↓invasion	Cheng et al. (2019b)
	Notch1/Jagged1/Hes-1 Signaling Pathway	PANC-1, and AsPC-1	↑↑ SNHG7: ↑stemness, and ↓ apoptosis SNHG7 regulates Folfirinox resistance in pancreatic cancer cells	Cheng et al. (2021)
Breast cancer	miR-15a	MCF7, and T47D	Δ SNHG7: ↓tumor cell proliferation, and ↓invasion	Li et al. (2020d)
	miR-34a	MCF-7, and MDA-MB-231	Δ SNHG7: ↑chemosensitivity of cancer cells to Adriamycin	Li et al. (2020a)
	miR-186	SK-BR-3, and AU565	Δ SNHG7: ↓ tumor cell proliferation, ↓migration and ↓EMT, and ↑apoptosis in chemoresistant cancer cells Δ SNHG7: ↑Trastuzumab sensitivity	Zhang et al. (2020b)
	miR-34a-5p/LDHA (Glycolysis) axis	MCF10A, MDA-MMB-436, HS578T, SKBR3, MDA-MB-231, and MCF-7	Δ SNHG7: ↓ tumor cell proliferation, and ↓glycolysis	Zhang et al. (2019b)
	miR-381	MCF-10A, ZR-75–1, HCC-1973, MDA-MB-231, and MDA-MB-468	Δ SNHG7: ↓tumor cell proliferation, ↓colony formation, and ↓invasion	Gao and Zhou, (2019)
	miR-34a/Notch-1 pathway	MCF-10A, MCF-7, MDA-MB-231, MDA-MB-157, and MDA-MB-435	Δ SNHG7: ↓tumor cell proliferation, and ↓invasion	Sun et al. (2019)
	miR-186	MCF-10A, MCF-7, MDA-MB-231 and SKBR3	Δ SNHG7: ↓tumor cell proliferation, and ↓invasion	Luo et al. (2018)
Colorectal cancer	miR-23a-3p/CXCL12 axis	SW480, LoVo, RKO, and HCT116	Δ SNHG7: ↓tumor cell viability, ↓proliferation, and ↓migration	Liu et al. (2020)
	miR-193b/K-ras/ERK/cyclinD1 axis	—	Δ SNHG7: ↓tumor cell proliferation, and ↑apoptosis	Liu et al. (2019)
	miR-34a/GALNT7/PI3K/Akt/mTOR pathway	FHC, caco2, SW480, SW620, Hct116, and LoVo	Δ SNHG7: ↓tumor cell proliferation, ↓migration, ↓invasion, ↓vasculogenic mimicry, ↓cell cycle progression, and ↑apoptosis	Li et al. (2018b)
	miR-216b/GALNT1 axis	FHC, SW480, SW620, LOVO, and HCT-116	Δ SNHG7: ↓tumor cell proliferation, ↓migration, ↓invasion and ↑apoptosis	Shan et al. (2018)
Gastric cancer	miR-34a/LDHA (Glycolysis) axis	HGC27, and AGS	Δ SNHG7: ↓ tumor cell viability and ↑chemosensitivity of cancer cells to cisplatin	Pei et al. (2021)
	miR-485-5p	HS746 T, HGC-27, SNU-1, AGS, and GES-1	Δ SNHG7: ↓tumor cell proliferation, ↓migration, and ↓invasion	Zhao and Liu, (2021)
	miR-34a/Snail/EMT axis	GES-1, MKN-45, SGC-7901, and N87	Δ SNHG7: ↓tumor cell migration, and ↓invasion	Zhang et al. (2020a)
	P15 and P16	GES-1, BGC823, MGC803, SGC7901, N87, and AGS	Δ SNHG7: ↓tumor cell migration, ↓colony formation, ↑apoptosis, and ↑cell cycle arrest	Wang et al. (2017b)
Bladder cancer	miR-2682-5p/ELK1/Src/FAK signaling pathway	T24, SW780, J82, UM-UC-3, 5637, and SE780	Δ SNHG7: ↓tumor cell proliferation, ↓migration, ↓invasion, and ↑apoptosis	Wang et al. (2020c)
	Bax, p21, and E-cadherin	SW780, T24, UMUC, and 5637	Δ SNHG7: ↓tumor cell proliferation, ↓invasion, ↑apoptosis, and ↑expression of Bax, p21 and E-cadherin proteins	Xu et al. (2019)
	Wnt/β-catenin pathway	SV-HUC-1, T24, 5637, 253 J, TCC, J82, and EJ	Δ SNHG7: ↓tumor cell proliferation, ↓colony formation, ↓migration, and ↑cell cycle arrest	Chen et al. (2019b)
	—	SV-HUC-1, T24, J82, and SW780	Δ SNHG7: ↓tumor cell proliferation, ↓invasion, ↓EMT, and ↑apoptosis	Zhong et al. (2018b)
Pituitary adenocarcinoma	miR-449a	GH1, RC-4B/C, GH3 and MMQ	Δ SNHG7: ↓tumor cell proliferation, ↓migration, and ↓invasion	Yue et al. (2021)
Glioma	miR-342-3p/AKT2 axis	A172, U87, U251, and SHG44	↑↑ SNHG7: ↑tumor cell proliferation, ↑migration, and ↑invasion	Cheng et al. (2020)
	miR-506-3p/CTNNB1 axis	NHA, U87, U251, SHG44, and A172	Δ SNHG7: ↓tumor cell proliferation, ↓colony formation, and ↑apoptosis	Du et al. (2020)
	miR-138-5p/EZH2 axis	LN229, A172, U251, and U87	Δ SNHG7: ↓tumor cell proliferation	Deng et al. (2021)
Glioblastoma (GBM)	miR-449b-5p/MYCN axis	NHA, T98G, U87, U251, and LN229	Δ SNHG7: ↓GBM cell viability, ↓migration, and ↓invasion	Chen et al. (2020b)
	miR-5095/Wnt/b-catenin pathway	HEB, A172, U87, T98G, and SHG44	Δ SNHG7: ↓tumor cell proliferation, ↓migration, ↓invasion, and ↑apoptosis	Ren et al. (2018)
Neuroblastoma	miR-323a-5p and miR-342-5p/CCND1 axis	SH-SY5Y, SK*-N-*SH, NB-1, SK*-N-*AS, and HUVEC	Δ SNHG7: ↓tumor cell migration, ↓invasion, and ↓glycolysis	Jia et al. (2020)
	miR-653‐5p/STAT2 axis	SK‐N‐AS, SK‐N‐SH, SH‐SY5Y, IMR‐32, and SK‐N‐BE Hombach and Kretz (2016) ‐C	Δ SNHG7: ↓tumor cell proliferation, ↓migration, ↓invasion, ↓EMT, ↑cell cycle arrest, and ↑apoptosis	Chi et al. (2019)
Ovarian cancer	EZH2/KLF2 axis	OC A2780, OCC1, H8710 and SK-OV3	Δ SNHG7: ↓tumor cell proliferation, ↓migration, ↓invasion, and ↓EMT	Bai et al. (2020)
Melanoma	six human UM cell lines	EZH2	Δ SNHG7: ↓tumor cell proliferation, ↑cell cycle arrest, and ↑apoptosis	Huang et al. (2020)
Cervical cancer	DKK1/Wnt/β-catenin axis	H8, C-33A, CaSki, SiHa, and HeLa	Δ SNHG7: ↓tumor cell proliferation, ↓colony formation, and ↑apoptosis	Chi et al. (2020)
	miR-485-5p/JUND axis	Ect1/E6E7, HEK-293T, Hela, SIHA, C-33A and HT-3	Δ SNHG7: ↓tumor cell proliferation, ↓migration, ↓invasion, and ↓EMT	Zhao et al. (2020)
	—	HeLa, and C-33A	Δ SNHG7: ↓tumor cell proliferation, and ↓invasion	Zeng et al. (2019)
Thyroid cancer	miR‐449a/ACSL1 axis	Nthy‐ori‐3–1, FTC133, TPC1, BCPAP, and 8505C	Δ SNHG7: ↓tumor cell proliferation, ↓migration, and ↑apoptosis	Guo et al. (2020)
	—	CAL62, and SW579	Δ SNHG7: ↓tumor cell proliferation, and ↓cell cycle	Chen et al. (2019c)
	BDNF	K1, TPC-1, SW579, and Nthy-ori 3–1	Δ SNHG7: ↓tumor cell proliferation, ↓colony formation, and ↑apoptosis	Wang et al. (2019)
	miR-9-5p/DPP4 axis	TPC-1, and B-CPAP	Δ SNHG7: ↓tumor cell proliferation, and ↓^131^I resistance	Chen et al. (2021)
Prostate cancer	miR-324-3p/WNT2B axis	RWPE, LNCaP, PC-3, and Du-145	Δ SNHG7: ↓tumor cell proliferation, ↓migration, ↓invasion, and ↓EMT	Han et al. (2019)
	miR-503/cyclin D1 axis	WPMY1, LNCaP, VCaP, 22RV1, DU145, and PC3	Δ SNHG7: ↓tumor cell proliferation, and ↓colony formation	Qi et al. (2018)
Osteosarcoma	p53/DNMT1 axis	U2OS, HOS, MG-63, and Saos-2	Δ SNHG7: ↓tumor cell proliferation, ↑cell cycle arrest, and ↑apoptosis	Zhang et al. (2019c)
	miR-34a	hFOB1.19, MG63, SaOS2, HOS, and 143B	Δ SNHG7: ↓tumor cell proliferation, ↓migration, ↓invasion, and ↓EMT	Deng et al. (2018)
	miR-34a-5p/*RAD9A* axis	GSE70415 dataset	*in situ* evaluations showed that SNHG7 may enhance cell proliferation and metastasis	Wang et al. (2020d)

Δ: knockdown or silencing, ↓: decrease or repression, ↑: increase or induction, ↑↑: overexpression, EMT: epithelial-to-mesenchymal transition.

Exosomal lncRNAs show high stability and concentrations and, thus, can be detected in body fluids (Tellez-Gabriel and Heymann, 2019). Regarding changes in expression levels of lncRNAs and their high diagnostic values, this makes them appropriate candidates for diagnosis and prediction of prognosis in human cancers (Qian et al., 2020). Several methodologies, including ultracentrifugation, are used to isolate exosomes and then detect the RNAs within. Although, due to low costs and higher accessibility, qRT-PCR is routinely used, high-throughput technologies such as next generation sequencing (NGS) and microarrays have facilitated detection of lncRNAs (Yamada et al., 2018). LncRNAs show acceptable values as diagnostic and prognostic biomarkers for several human cancers (Qian et al., 2020). In this review, we outline the cellular, animal, and clinical studies indicating that this lncRNA is almost universally upregulated in cancer tissues, promotes malignant features of cancer cells, and has prognostic value in various malignancies; however, it seems that SNHG7 diagnostic accuracy in discrimination of human malignancies requires further investigation. Additionally, major limitations of detection methods, such as the impossibility of detecting the amplicon size, limit the number of lncRNAs that can be simultaneously detected, and nonspecific binding, which restricts the clinical application of commonly used qRT-PCR, requires more time to take the lncRNAs into the clinical setting (Jensen, 2012). Finally, there is no CRISPR-based genome editing or siRNA-based method approved or tested for suppression of SNHG7.

In conclusion, regarding a considerable number of studies that reveal oncogenic role of SNHG7 in human cancers and its prognostic value, SNHG7 is suggested as a potential cancer biomarker for human malignancies. Further investigations and more time are required for SNHG7 clinical applications in detection, prediction of prognosis, and treatment of human malignancies.
